# New Mitochondrial Targets in Fungal Pathogens

**DOI:** 10.1128/mBio.02258-19

**Published:** 2019-10-01

**Authors:** Daniel Murante, Deborah A. Hogan

**Affiliations:** a Geisel School of Medicine at Dartmouth, Hanover, New Hampshire, USA

**Keywords:** *Candida albicans*, *Candida* species, mitochondria

## Abstract

In eukaryotic cells, mitochondria are responsible for the synthesis of ATP using power generated by the electron transport chain (ETC). While much of what is known about mitochondria has been gained from a study of a small number of model species, including the yeast Saccharomyces cerevisiae, the general mechanisms of mitochondrial respiration have been recognized as being highly conserved across eukaryotes. Now, Sun et al. (N. Sun, R. S. Parrish, R. A. Calderone, and W. A. Fonzi, mBio 10:e00300-19, 2019, https://doi.org/10.1128/mBio.00300-19) take the next steps in understanding mitochondrial function by identifying proteins that are unique to a smaller phylogenetic group of microbes. Using the combination of *in silico*, biochemical, and microbiological assays, Sun and colleagues identified seven genes that are unique to the CTG fungal clade, which contains multiple important human pathogens, including Candida albicans, and showed that they are required for full ETC function during respiratory metabolism. Because respiratory metabolism is critical for fungal pathogenesis, these clade-specific mitochondrial factors may represent novel therapeutic targets.

## Abstract (precis)

In eukaryotic cells, mitochondria are responsible for the synthesis of ATP using power generated by the electron transport chain (ETC). While much of what is known about mitochondria has been gained from a study of a small number of model species, including the yeast Saccharomyces cerevisiae, the general mechanisms of mitochondrial respiration have been recognized as being highly conserved across eukaryotes. Now, Sun et al. (N. Sun, R. S. Parrish, R. A. Calderone, and W. A.

## COMMENTARY

Invasive candidiasis affects approximately 700,000 individuals worldwide on a yearly basis and has a mortality rate of 19 to 24% (1). Three of the major agents of candidiasis, Candida albicans, Candida tropicalis, and Candida parapsilosis, are members of the CTG phylogenetic clade of fungi, which is so named because they generally translate CTG as serine rather than leucine. The CTG clade also contains Candida lusitaniae, an emerging opportunistic pathogen in immunocompromised hosts, and Candida auris, which has recently been the cause of candidiasis outbreaks and is problematic due to its multidrug resistance (2). The CTG codon reassignment has been previously established to have occurred approximately 170 million years ago, ample time for divergence in mitochondrial function. Furthermore, the codon ambiguity itself is predicted to dynamically affect the function of proteins and thus may drive evolution in these species (3, 4).

Electron transport chain (ETC) function in the mitochondria impacts multiple aspects of pathogenesis, particularly growth, antifungal susceptibility, the host immune response, and the yeast-to-hypha transition (5). As a result, the fungal mitochondrion is a potential focus for therapy, and the identification of mitochondrial targets is of great importance. However, many genes are conserved between yeast and human mitochondria, complicating drug development (6).

To identify putative mitochondrial genes specific to a clinically important phylogenetic clade of fungi, Sun and colleagues (7) conducted an *in silico* comparison of protein sequences in C. albicans to those in Saccharomyces cerevisiae and Aspergillus nidulans, to establish a subset of 1,349 poorly conserved C. albicans proteins. This subset was then further analyzed to establish a subset of proteins found in all CTG clade members. Among these, proteins predicted to localize to the mitochondria were selected for identification of those with no homologs outside the CTG clade. The *in silico* analysis by Sun et al. (7) yielded 25 genes of interest, 23 of which had not been previously characterized. This analysis also found *GOA1*, a protein that had been previously identified as being unique to CTG clade fungi (8), validating their methodology. Seven of these uncharacterized CTG clade-unique genes were required for full respiratory activity.

The mitochondrial respiratory chain is composed of five large multisubunit enzymes, complexes I to V, embedded within the inner mitochondrial membrane (9). NADH and ubiquinone donate electrons to the ubiquinone pool via complex I and complex II, respectively. Complex III funnels electrons from the ubiquinone pool to cytochrome *c*, and then complex IV ultimately transfers the electrons to oxygen. These processes generate a proton gradient that is used to generate ATP by complex V, ATP synthase. Of the seven gene knockouts that diminished respiratory function, deletion of *NUO3*, *NUO4*, *NUE1*, or *NUE2* impaired complex I function. Deletion of *QCE1* resulted in a loss of complex III, and mutation of *COE1* or *COE2* resulted in decreased complex IV activity. *MNE1* was also identified in this screen, and its expression was necessary for the normal function of complex I. Although it has a homolog in S. cerevisiae, *MNE1* demonstrated a case of functional reassignment, given that it is involved in the expression of complex III in S. cerevisiae and complex I in C. albicans. The finding of new components in three different mitochondrial complexes highlights the breadth of changes that occurred over evolutionary time. All of these genes also played an important role in respiratory metabolism in pathogenesis, as shown by virulence attenuation of these mutants in Galleria mellonella.

The identification of CTG clade-unique genes involved in ETC function raises the possibility of new therapeutic targets for C. albicans, C. tropicalis, and C. parapsilosis, prominent agents of invasive candidiasis in the world today, as well as other members of the CTG clade. As a rising global health threat and often multidrug-resistant member of this clade, C. auris in particular highlights the potential utility of more targeted therapeutics. Researching compounds to target these genes with CTG clade specificity may lead to the development of well-tolerated yet narrow-spectrum antifungals and could be an effective way to circumvent off-target toxicity.

These findings give rise to several unanswered questions. While the CTG clade specificity of these genes may make for attractive potential therapeutic targets, another fascinating area of study is why these genes are necessary for the CTG clade and not for non-CTG clade fungi. Do these proteins have roles that are specific to mitochondrial metabolism, or do they have a broader role in fungal physiology? The lack of these genes in Candida glabrata, an important agent of candidiasis that is not in the CTG clade, indicates that these proteins are not absolutely required for pathogenesis. Clade-specific changes in highly conserved genes may reveal deeper insights into their broad functional roles and perhaps inform larger differences in metabolism between taxonomic groups. Furthermore, these studies suggest that it may be fruitful to consider whether broadly conserved ETC proteins have additional roles that also require these newly discovered mitochondrial components. In addition, the seven previously uncharacterized genes identified in this paper may also have important functions that are evident only under certain conditions. As an example, Goa1 localizes to the mitochondria during stress and is potentially regulated by Rbf1, a repressor of hyphal morphology (5, 10). Transcriptional analysis of the genes identified in this paper as well as the identification of their transcriptional regulators will provide more information on when their functions are most important.

In summary, the work by Sun et al. (7) highlights the underappreciated fact that there are differences in the mitochondria of fungal pathogens compared to the model yeast S. cerevisiae. An important future direction of this research would be the identification of proteins unique to other fungal clades to look for the conserved mitochondrial components in non-CTG species, and this analysis might provide insight into the functions of clade-specific mitochondrial proteins. Such studies may elucidate traits that drive evolution of these species and how the clade-specific ETC factors affect fungal physiology. This knowledge will be critical for the thoughtful development of new antifungal strategies.
